# The land and sea routes to 2030: a call for greater attention on all small islands in global environmental policy

**DOI:** 10.1038/s44185-023-00023-5

**Published:** 2023-09-07

**Authors:** Andrea Monica D. Ortiz, Ma. Laurice Jamero, Silvio Javier Crespin, Cecilia Smith Ramirez, Denise Margaret S. Matias, Jameela Joy Reyes, Aníbal Pauchard, Antonio G. M. La Viña

**Affiliations:** 1 Institute of Ecology and Biodiversity (IEB), Victoria 631, Barrio Universitario, Concepción, Chile; 2 Manila Observatory, Quezon City, Philippines; 3 Laboratorio de Estudios del Antropoceno, Universidad de Concepción, Concepción, Chile; 4 Instituto de Investigaciones Tropicales de El Salvador, San Salvador, El Salvador; 5 Departamento de Ciencias Biológicas y Biodiversidad, Universidad de Los Lagos, Osorno, Chile; 6 Eberswalde University for Sustainable Development (HNEE), Eberswalde, Germany; 7 Ateneo de Manila University, Quezon City, Philippines; 8 Laboratorio de Invasiones Biológicas (LIB), Facultad de Ciencias Forestales, Universidad de Concepción, Concepción, Chile

**Keywords:** Climate change, Policy, Biodiversity, Conservation biology, Invasive species, Ecology, Climate-change ecology, Conservation biology, Ecosystem services, Invasive species

## Abstract

Islands have unique vulnerabilities to biodiversity loss and climate change. Current Nationally Determined Contributions under the Paris Agreement are insufficient to avoid the irreversible loss of critical island ecosystems. Existing research, policies, and finance also do not sufficiently address small islands’ social-environmental challenges. For instance, the new Global Biodiversity Framework (GBF) mentions islands in the invasive species management target. This focus is important, as islands are at high risk to biological invasions; however, this is the only GBF target that mentions islands. There are threats of equal or greater urgency to small islands, including coastal hazards and overexploitation. Ecosystems such as coral reefs and mangroves are crucial for biodiversity, coastal protection, and human livelihoods, yet are unaddressed in the GBF. While research and global policy, including targeted financial flows, have a strong focus on Small Island Developing States (SIDS), the situation of other small islands has been largely overlooked. Here, through a review of policy developments and examples from islands in the Philippines and Chile, we urge that conservation and climate change policies place greater emphasis on acknowledging the diversity of small islands and their unique governance challenges, extending the focus beyond SIDS. Moving forward, global policy and research should include the recognition of small islands as metacommunities linked by interacting species and social-ecological systems to emphasize their connectivity rather than their isolation. Coalition-building and knowledge-sharing, particularly with local, Indigenous and traditional knowledge-holders from small islands, is needed to meet global goals on biodiversity and sustainable development by 2030.

## Introduction

Climate change threatens the existence of all islands globally through sea level rise and extreme weather events. Biodiversity loss is also a major issue on islands, which are home to 20% of the world’s biota, even though they comprise only 6% of total land area^1^. Endemic island species are inherently vulnerable due to genetic and demographic factors, in addition to being threatened by human activities, invasive species, and land/sea-use change^1,2^. Islands are also hotspots of biocultural heritage and knowledge^3^. While islands vary greatly in size, geology, and history, Small Island Developing States (SIDS) are often the focus of science, policy, and the media as frontlines for global environmental crises. This focus has led to mainstream research on small islands; however, it has also resulted in biases in risk interpretation, and less attention on other vulnerable dependent islands and subnational island jurisdictions^4^. [Box 1].

Currently, the heterogeneity of islands is not reflected in major environmental policy frameworks of the United Nations Framework Convention on Climate Change (UNFCCC) and Convention on Biological Diversity (CBD), which have a strong focus on SIDS. It is a fact that SIDS are some of the most exposed and vulnerable communities to environmental change^5,6^. Their leadership in policy platforms has placed them at the forefront of ambition to address the global environmental crises. At the same time, other small islands beyond SIDS remain out of focus from global policy and scientific discourse. This is alarming, as these other small islands share many of the vulnerabilities of SIDS, but lack their platform and coalition for influencing global policy.

Many of the outcomes of the recent Conferences of the Parties of the UNFCCC and CBD (COP27 and COP15 in 2022, respectively) have been lauded as significant progress, notably on issues such as Loss and Damage, and the new Kunming-Montreal Global Biodiversity Framework (GBF) for stopping biodiversity loss by 2030. Here, we ask: how much of these policies focus on vulnerable human and natural communities on all small islands, particularly those in developing countries?

To address this question, we summarize current threats to island ecosystems and communities, and examine how well recent global policy developments reflect the different needs of small islands. We highlight examples from Chile and the Philippines, two countries with over 51,000 islands in total, to illustrate how their archipelagic geographies affect their vulnerability. We discuss how the unique governance and conservation challenges of small islands in developing countries impede a proper distribution of resources and a timely uptake of action against climate change and biodiversity loss. From our experiences as climate scientists, ecologists, and negotiators, we reflect upon how and where we can improve to achieve the targets of the Agenda for Sustainable Development and GBF by 2030.

Box 1 Types of islands, based on physical characteristics and political groupings Islands can be classified based on their geological origins, such as volcanic islands, (coral reef) atolls and barrier islands, continental islands separating from larger landmasses, and composite islands which are both volcanic and tectonic in origin (See also in refs. ^1,2^). There are also political island categories: SIDS, subnational island jurisdictions (SNIJ) and dependent islands^4^. SIDS were formed at the 1992 Rio Earth Summit as a response to shared challenges and vulnerabilities, at present counting 39 members and 18 associate members; the latter are non-UN island territories of developed countries^6^. A number of SIDS are also least developed countries, but the diverse and non-homogeneous group also counts coastal non-island nations such as Suriname, Guyana, and Belize. SNIJ are semi-autonomous islands that exhibit significant legislative competence and executive governance^51^. Dependent islands are part of continental states or larger island states with no particular degree of autonomy^4^.

## Results

### Threats to island ecosystems and natural communities

**Fig. 1:**
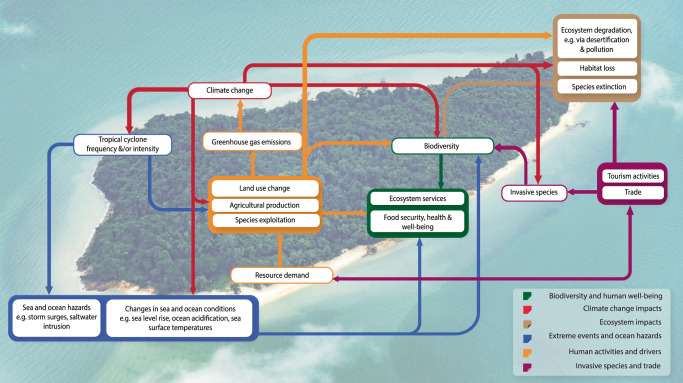
The nexus of interactions in small island systems. Anthropogenic activities (orange arrows) such as land use change contribute to climate change, which itself exacerbates these impacts (red arrows). Climate change, invasive species (magenta arrows) and extreme events such as tropical cyclones (blue arrows) also have significant impacts on biodiversity. Feedbacks between these factors will affect the provision of ecosystem services toward human communities (green arrows), as well as threaten unique island species and ecosystems (brown arrows). There are many other important factors in this nexus, such as population growth, affluence, and policy agreements, which all have impacts on biodiversity in an intricate and dynamic system^11^, reflecting the complexity of island development and conservation. Here, we focus on a simplified representation of this complex system to illustrate the connectedness within island social-ecological systems. Background image was obtained from Pixabay on a CC0 license.

As biodiversity is essential for health and food security, its loss will have profound impacts on human populations on small islands. This is of significant concern because the limited genetic diversity island species limits their potential for adaptation to changing environments. The physical geography of islands also restricts their ability to shift in latitude or altitude to respond to climate change^7^. Sea level rise can affect infrastructure and settlements in low-lying coastal zones^2,8^. Climate change and coastal hazards like saltwater intrusion reduce habitat availability, suitability, and already limited freshwater resources^9,10^. Islands’ physical characteristics also influence risks: for example, atoll islands are vulnerable to seawater infiltration due to the permeable nature of their island substratum^8^. These interactions add to the complex dynamics of island social-ecological systems (Fig. 1).

Land use change, resource over-extraction and invasive species affect native species and nature’s contributions to people that are essential for human well-being. Other important factors include population growth, affluence, and international trade, which all affect biodiversity in a complex nexus^11^. For example, international trade is a key driver of invasive species introductions^12,13^. Invasive alien species (IAS) are a major driver of species extinctions on islands^14^ and a major threat to marine life^15^. However, many island populations are dependent on international trade, and may have tourism-focused economies^16^. Globalization has also led to a breakdown of biogeographical barriers, contributing to island vulnerability to IAS^13^.

These impacts are all compounded by climate change, which disrupts and constricts species’ ranges and distributions^17^. Climate change also causes decreases in sea oxygen concentration, rises in sea surface temperature, and increases in ocean acidification that affect marine biodiversity^18^. Extreme events like tropical cyclones and their associated hazards such as storm surges affect not only people and assets, but also species and ecosystems that support livelihoods, services and products that billions of people depend on^19^. Tropical island systems like the Philippines are exposed and vulnerable to these powerful hazards^20^, which are anticipated to increase in intensity^21^.

### Recent policy developments and their implications for small islands: loss and damage

In this section, we discuss the implications of recent advances in global climate change and biodiversity policy in the context of small islands, and the effects of the focus on SIDS in these discussions.

Loss and damage (L&D) is a critical issue for all small islands. In the UNFCCC, progress would not have been made without the collective leadership of SIDS. The island nation of Vanuatu, an archipelago part of the Alliance of Small Island States (AOSIS), was the first to propose an insurance scheme to provide financial resources to countries affected by sea level rise when the UNFCCC was being crafted in 1991. At the UNFCCC COP21 in Paris (2015), AOSIS, the Group of 77 and China, and the Climate Vulnerable Forum were influential on keeping a target of a global mean temperature change of 1.5 °C above pre-industrial levels^22^. Vanuatu also led a successful petition for the International Court of Justice to render an advisory opinion on the legal obligations of developed country States, particularly towards SIDS^23^.

More than three decades of advocacy culminated in the landmark decision at the UNFCCC COP27 in 2022 to create the Loss and Damage Fund (LDF). Rightfully celebrated as a triumph, the LDF will provide technical support and financial assistance to avert, minimize, and address losses and damages associated with the adverse effects of climate change in ‘particularly vulnerable’ developing countries^24^. L&D is divided into two main categories: (1) economic L&D affecting resources, goods, and services that are normally traded in markets and (2) non-economic L&D, which are understood as losses and damages to individuals (e.g., to life, health, and human mobility), to society (which includes health, cultural heritage, indigenous knowledge, and societal and cultural identity), and to the environment (including to biodiversity and ecosystem services).

Considering that over half of the world’s Gross Domestic Product, about $44 trillion, is dependent on nature^25^, it is paradoxical that the environment is considered only under non-economic L&D and not both. More significantly, nature has diverse values beyond its economic value. An important driver of the global decline of biodiversity is the unsustainable use of nature based on decisions made from a narrow set of values, such as those traded in markets^26^ – precisely what L&D is anticipated to compensate for. Due to difficulties surrounding their valuation, discussions regarding compensation for non-economic L&D are exceptionally challenging. How will parties approach compensation for the loss of biodiversity on small islands, including cultural ecosystem services and relational values linked to nature? These remaining ethical and moral dilemmas signal critical work that remains to be done to deepen our understanding of nature’s diverse values.

Principles of the Multiple Evidence Base can be applied when determining compensation for non-economic L&D to recognize that each relevant actor has their own interests that influence their valuation of nature^27^. The Santiago Network established at UNFCCC COP25 must inclusively identify technical needs and priorities, respecting diverse valuations and values of nature. Importantly, policymakers need to ensure that compensation covers what future generations have already lost, and will never recover.

L&D becomes a matter of survival when considering the current state of Nationally Determined Contributions (NDCs) following the Paris Agreement. Even if current NDCs are implemented, emissions are projected to lead up to 3.2 °C of warming^28^. These changes will affect human lives profoundly. At 1.5 degrees, 9% of the world’s population will be exposed to extreme heatwaves at least once every 20 years, and this triples to 28% (2 billion people) with half a degree more^28^. Unless urgent actions are instigated, people will face significant water and food insecurity. Immediate and deep reductions of greenhouse gases beyond what are pledged in NDCs are urgently needed. If the tremendous expectations of the LDF are fulfilled, it may spell the difference for small islands and other vulnerable developing countries by awarding rightful compensation.

### Islands in the Global biodiversity framework: tackling invasive species and other threats

In addition to being sensitive to climate change, island ecosystems and species are vulnerable to IAS, land use change, and overexploitation^1^. The new GBF includes Target 6 on IAS, which explicitly mentions islands as priority sites: “…reducing the rates of introduction and establishment of other known or potential invasive alien species by at least 50%, by 2030, eradicating or controlling invasive alien species especially in priority sites, such as islands”^29^. This goal is particularly welcome, considering the vulnerability and risk of islands to species extinctions^13^. An example are volcanic Chilean islands like the Juan Fernández Archipelago affected by IAS [Box 2].

The recognition of the threat posed by IAS on islands in the GBF is a major step forward. However, while acknowledging the significant progress represented by GBF’s Target 6, it is essential to emphasize that other threats of equal or greater urgency exist within island ecosystems. In fact, there is great variability in the main threats to insular flora and fauna globally^7,30^. This is one area where the GBF falls short in acknowledging the diverse needs of islands. Target 6 is the only target that explicitly mentions islands as priority sites for biodiversity.

The GBF fails to mention coral reefs, which are only considered broadly under Target 8, which is to “minimize the impact of climate change and ocean acidification on biodiversity”^29^. The loss of coral reefs is a core “climate tipping point”; coral collapse under 1.5 °C of warming would remove one of the Earth’s most biodiverse ecosystems, affecting the marine food web, ocean nutrient and carbon cycling, and livelihoods of millions of people^31^. Coral reefs and other coastal ecosystems such as seagrass and mangrove ecosystems protect coastal communities from wave erosion, tropical cyclones, and storm surges^2^. With current NDCs deemed grossly insufficient to avoid dangerous warming^28^, much is lacking in the GBF to acknowledge the vulnerability of these critical coastal and island ecosystems.

In addition, there are key differences with how SIDS and other small islands are addressed in the GBF. SIDS are mentioned specifically in Target 19 and Goal D of the GBF as targeted recipients of increased funding flows, capacity building, and technology transfer from developed countries. These funds are hoped to reach at least US$20 billion per year by 2025, and to at least US$30 billion per year by 2030^29^. In order to fully address biodiversity loss, the scope of the GBF and the resource prioritization needs to include all small islands, including and beyond SIDS. Likewise, the focus on IAS urgently needs to be expanded to include other critical threats to biodiversity on small islands.

**Fig. 2:**
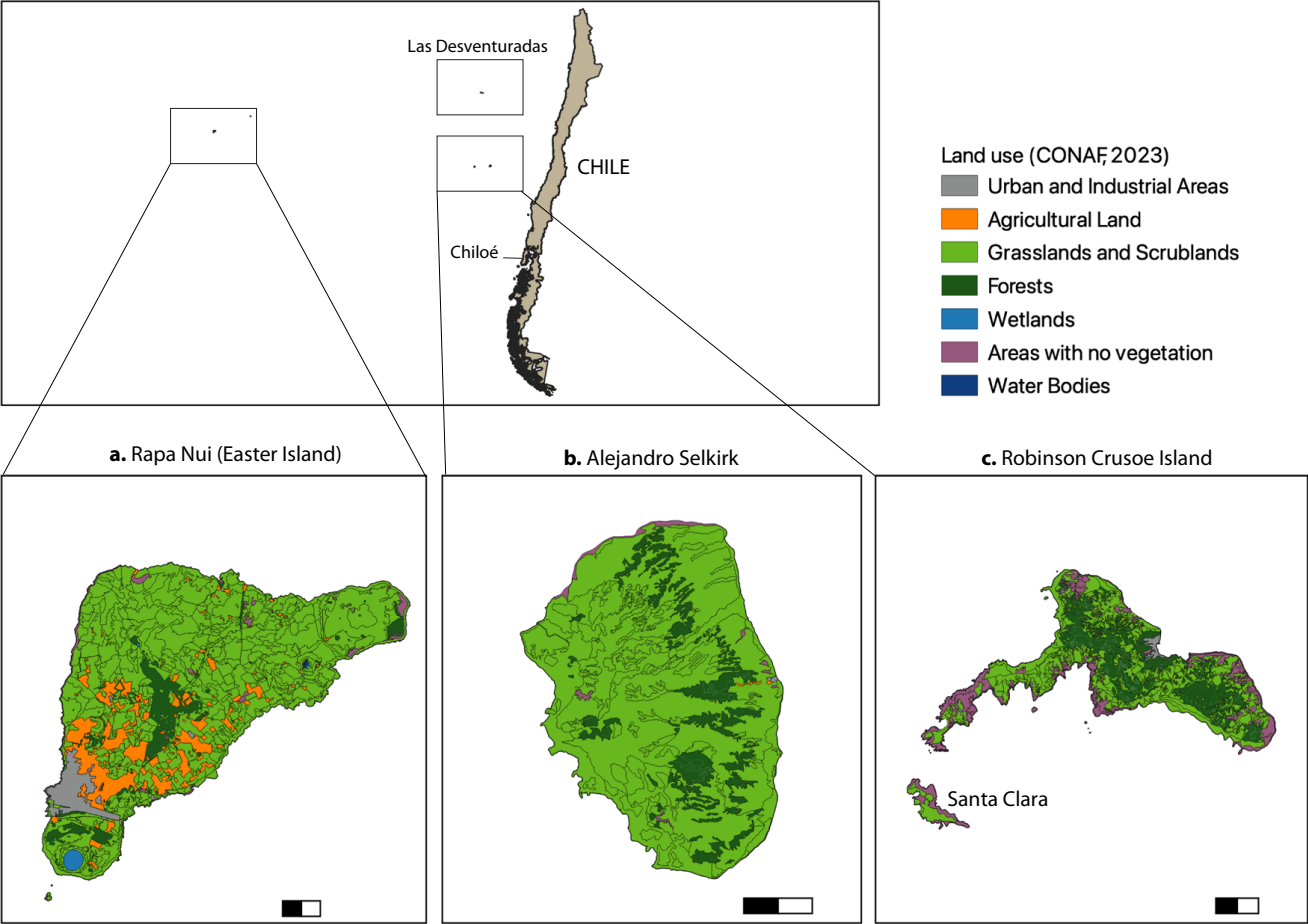
Chile’s island territories. Chile counts several islands and archipelagos as part of its national territory. Rapa Nui (Easter Island), and the islands of the Juan Fernández Archipelago (the islands of **a** Robinson Crusoe, **b** Alejandro Selkirk and **c** Santa Clara) are threatened by invasive species and overexploitation, which can decimate endemic biodiversity as is the case of Rapa Nui. Robinson Crusoe Island (**c**) is globally the fourth most invaded surface by woody plants. Vegetation cover varies throughout the islands, but little forest cover remains in Rapa Nui^50^. Scale bar shows 1 km.

Box 2 Invasive alien species management in Chilean island territories Chile counts several oceanic islands as part of its territory. These include the Juan Fernández Archipelago (JFA), Rapa Nui and Las Desventuradas (Fig. 2). Robinson Crusoe Island (RCI) is part of the JFA composed of three volcanic islands in the Pacific Ocean. The late scientist E.O. Wilson referred to this archipelago as islands of the “living dead”, due to the high quantity of critically endangered species^52^. JFA is located 470–800 km in front of Chilean central coast, and is considered one of the two smallest biodiversity hotspots globally – the Galapagos Islands being the other – because of its extraordinarily high and ancient endemism^53^. The most studied island of the JFA is RCI, which is globally the fourth most invaded island in surface area by woody plant species^54^. The expansion of invasive woody species, mainly common bramble (*Rubus ulmifolius)*, maqui (*Aristotelia chilensis)*, strawberry myrtle (*Ugni candollei)* and other species from the *Cupressus* and *Pinus* genera, was estimated at 4.3 ha annually in 2014^54^.

These IAS are established under forest canopy gaps, out-competing endemic species. Only three remaining forest fragments remain on the island, with each containing no more than 40 hectares^55^. The control of invasive plant species has been made mainly by the non-governmental organization OIKONOS, which is focused on small gaps in one forest. Their efforts show small but effective success^56^. However, the rate at which effective chemical control occurs compared to the rate at which invasive plant species advance is insufficient to prevent biodiversity loss.

In another island of JFA, Santa Clara, spontaneous succession after rabbit eradication is slowly and successfully occurring^57^. The third island of the archipelago, Alejandro Selkirk, is highly threatened by wild goats and there are no policies to eradicate them. In general, the Chilean government does not give adequate importance to the loss of the unique biodiversity of these islands^57^.

Another island of significance in Chile is Rapa Nui (Easter Island or *Isla de Pascua*), also in front of the Chilean coast, threatened not by IAS expansion but by the deforestation that occurred several centuries ago before the arrival of occidental civilization. Only pollen records remain of what was probably a rich and endemic biodiversity. Deforestation and habitat loss were extreme on this island. Only some species of ferns, native herbs, and some native insects remain in a volcanic cave^58^. There are no studies of the effect of climate change in these islands but both are exposed to intense erosion^59^, and subject to an increase of coastal hazards due to the intensity of atmospheric rivers combined with sea level rise^60^.

As there are no specific governance strategies for islands in Chile, we argue that it is necessary that regulations on carrying capacity and protecting ecosystem services should be put in place as well as the implementation of restoration activities and research, for example on native ferns that appear to inhibit invasion in RCI^61^. The history of overexploitation and ecological disasters that are happening on Rapa Nui is an important indication of what could happen on other islands in Chile.

### Conservation and governance challenges for small islands

The examples shown here indicate that at the global scale, and even within archipelagic countries, there is inadequate awareness of the complex challenges that small islands face. Because of their peripheral nature relative to their respective mainland or main island, small islands are neglected systematically in the development planning system. Many islands are already socially- and ecologically marginalized, so the lack of priority for them within local development planning equates to a form of double marginalization.

For instance, in the Philippines, island provinces are disadvantaged compared to provinces closer to the capital, based on economic, poverty, and human development indicators. Their reliance on Internal Revenue Allotments (IRA), which is the local provincial budget based on population size and land area, contributes to their dependence on the centralized state. This dependency hampers local growth, exacerbating the poverty experienced by island provinces^32^. These challenges are comparable to those of island nation-states, including vulnerabilities associated with geographic remoteness, limited economic diversification, susceptibility to natural hazards, and dependence on tourism^32^. These challenges arising from mainland relations are particularly important to recognize. Small islands often face governance issues that complicate their situation, such as the case of Danajon Bank [Box 3].

Mainland relations are also an issue in Chile, a country characterized by its continental, archipelagic, and Antarctic zones^33^. The Chiloé archipelago in Southern Chile (See Fig. 2) has experienced significant social and environmental damage due to the salmon industry. Its island identity and “islandness” (see ref. ^22^), shaped by collective identities related to the sea and relationship to the continental state, play a crucial role in understanding its social-ecological challenges^34^. The Chilean state’s support for the expansion of the salmon industry led to the exclusion of the *Chilotes* (people of Chiloé) from decision-making processes concerning the environment and economy. Although their proximity has offered some social and economic mobility, the insularity of archipelagos in South Chile (e.g. Chiloé, Calbuco) still remains. The consequences of historic isolation remain in island cultures and customs^35^. This is also evident in the genetic isolation of the threatened pudu (*Pudu puda)*, the smallest deer in the world, on Chiloé compared to continental Chile^36^.

**Fig. 3:**
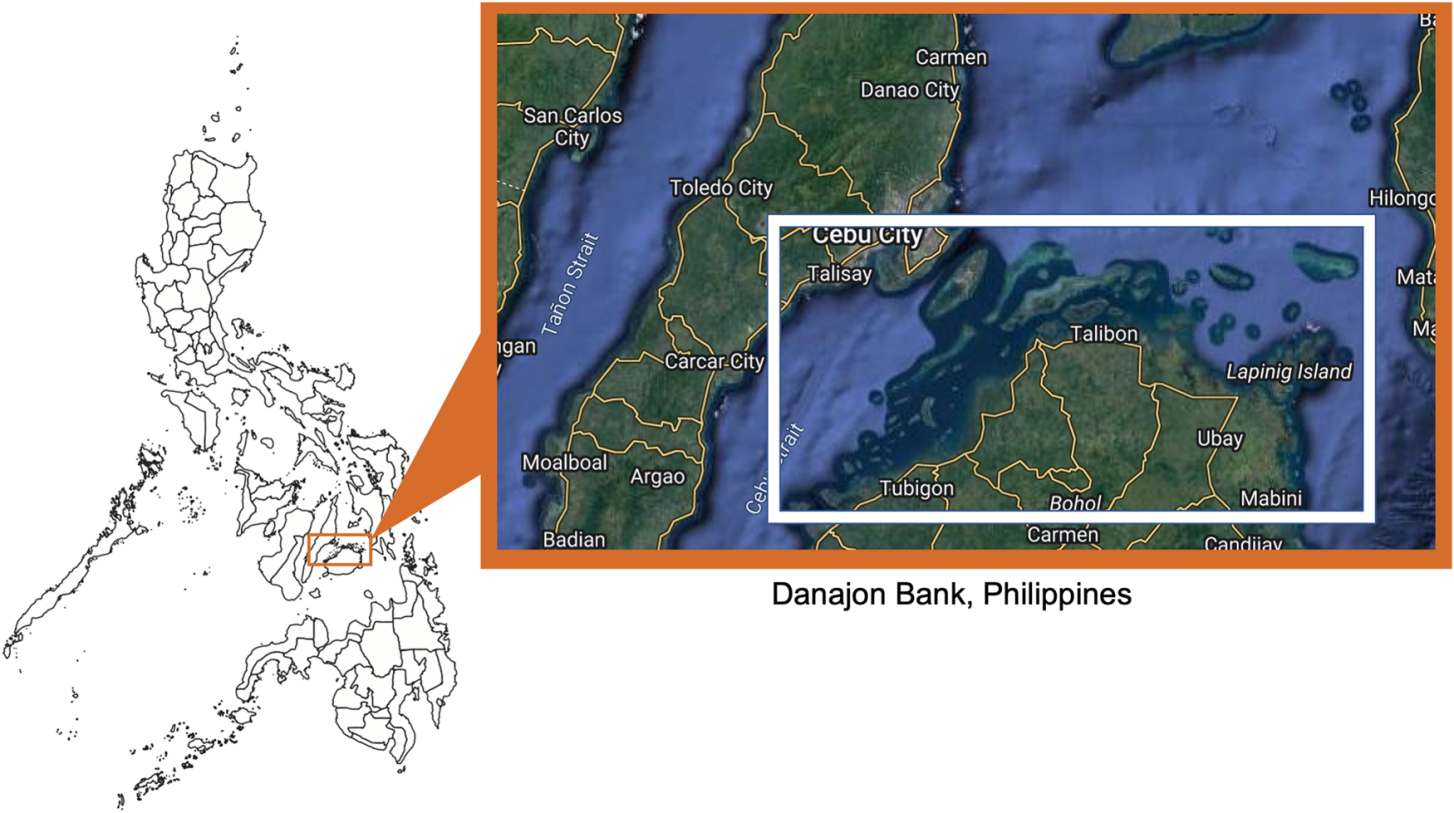
Double-barrier reef Danajon Bank, Philippines. Danajon Bank is spread across several island municipalities in central Philippines. Inset photo from Google Earth.

Conservation on small islands necessitates a delicate balance of multiple social-environmental objectives. Conservation challenges on small islands are closely linked to how local governance systems are adaptive to their unique needs. Current conservation strategies to protect areas of ecological importance are typically based on classical ecological theory, notably island geography studies (see ref. ^37^). In contrast, analyses of global spending show that island conservation efforts are underfunded relative to continents^3^. In the face of limited resources and the urgency to address climate change and biodiversity loss, what can help improve island conservation efforts?

We suggest that by incorporating insights from research on island typology, including political island groups (see Box 1, and refs. ^1,2^) we can better understand the intricate relationship between physical island characteristics, ecological processes, and human activities. Evolutionary processes are also key to understanding IAS on oceanic islands^38^. In addition, ecological processes, such as species migration and extinction, are already being affected by increasing climate velocities, especially those taking place in tropical coastal waters^39^. Many inconspicuous but important island species, such as vascular plants, arthropods, mollusks, and less-known invertebrates have already been driven extinct by human activities on islands^1^.

Importantly, island governance processes should be seen as key factors in mitigating risks and adapting to environmental change. Here, factors such as island sovereignty, mainland relations and access to resources become crucial (see Boxes 2 and 3). Therefore, a key action is to approach conservation from a metacommunity perspective, in addition to recognizing islands as social-ecological systems (e.g., in refs. ^40,41^). A metacommunity is “a set of local communities that are linked by dispersal of multiple potentially interacting species”^42–44^, meaning each island is a community in itself. Proximate islands and archipelagos should be ideally managed as a singular or, at the very least, a connected system.

To summarize these different considerations, we describe five key factors, adapted from the work of Sims et al.^41^ on social-ecological systems^41^: ecological and social heterogeneity, ecological and social spillovers, and uncertainty (Table 1).

**Table 1:** Important factors for consideration in developing conservation strategy in small islands (adapted from ref. ^41^).

1. Ecological heterogeneity	The quality and quantity of distinct resource types varies between islands. Properly informed conservation policy will require monitoring to build baselines throughout the multi-island system. Rather than a blanket policy, successful island governance will require the assessment of the conservation status of each island’s biological community to be made available to decision makers.
2. Social heterogeneity	Local island communities differ in the structure and strength of their economies, and the beliefs and values of their peoples. These differences affect a number of socio-economic and cultural aspects, including livelihoods, identities, and trade. Policies that do not take island diversity at the social level into account might face great challenge and opposition from local communities. National-level policies would benefit from increased alignment with local needs.
3. Ecological spillovers	In population dynamics, ecological spillovers can lead to increases in abundances beyond what resource limits can dictate, leading to species with the dispersal capability to colonize other islands^46^. In the same vein, governance actions on one island can lead to changes in another since they form interlinked social-ecological systems^40^. Conservation actions may lead to unplanned consequences. For example, hasty restoration of mangroves on islands and coastal areas of Eastern Samar in the Philippines after Typhoon Haiyan (2013) used the less laboriously cultivated but higher-mortality *Rhizophora* mangroves instead of the naturally-dominant species *Sonneratia alba* and *Avicennia marina*. Typhoon survivors began clearing defoliated (but live) trees and surviving seedlings to avail of cash grants^47^. Many post-Haiyan rehabilitation initiatives continue to use *Rhizophora* propagules and encroach on other sensitive coastal habitats like seagrass meadows across different islands^48^. In line with the precautionary principle, contingencies should be taken into account when planning ecological restoration in order to avoid unfavorable outcomes.
4. Social spillovers	These can occur when management actions on one island affect the cultural, human, physical, and natural capital of other islands. Island distribution can affect available amenities and transport systems; thus, the economy, human health, livelihoods and, cultural aspects of local communities might already be significantly different^49^ where changes can quickly cascade from one island community to another. This means that actions can also have impacts beyond a single island community.
5. Uncertainty and irreversibility	Stochastic events are a property of all social-ecological systems, and interconnected small island systems are no exception. However, because of the unique traits and small size of ecological communities on island systems, they are especially vulnerable to unforeseen catastrophic events. The challenge for governance lies in development planning that accounts for these uncertainties, such as in disaster risk reduction and climate change adaptation.

Box 3 The case of Danajon Bank, Philippines Danajon Bank is a double barrier reef, one of only six in the world, spread across coastal municipalities in the island provinces of Bohol, Leyte, Southern Leyte, and Cebu in the Philippines (Fig. 3)^62^. Despite being a biodiversity hotspot, Danajon Bank is not managed as a singular ecosystem. Islets within this ecologically-important marine area are governed separately based on administrative boundaries, where different local government units (LGUs) are in charge of different islets^63^. Danajon Bank supports coral reef, soft bottom, and seagrass habitats^64^.

Because of their distance at sea, the islets in Danajon Bank are not easy to access, especially during the rainy monsoon season. Based on experience, many LGU officials are unable to make regular visits to the local island communities. The islets are only a few hectares wide and support a few hundred households. Their size also limits their IRA, the local budget allocation. While there are some positive developments to govern the Bohol side of Danajon Bank, such as the establishment of a council and other project interventions from different agencies^62^, the lack of an ecosystem-wide approach to managing Danajon Bank has led to a “tragedy of the commons”.

The double barrier reef suffers from overfishing, illegal fishing, and decreasing fish stocks aggravated by the impacts of storm surges, sea level rise, and ocean acidification. Coral reefs are threatened by mass bleaching, coral mining, reclamation, and in some areas, poor management of tourism, which also affects coral reefs. The Danajon Bank communities in Bohol are heavily reliant on fishing and resource extraction, mostly for subsistence. There are major concerns on access to water, sanitation, and fuel in the area^62^. While this poverty and lack of access to alternative skills are commonly cited reasons for small-scale fishers continuing to fish in overexploited environments, there are also many complex social and community factors that influence fisherfolk’s actions that affect ecosystems in the area^65^.

Efforts for establishing a network of community-based marine protected areas (MPAs) within Danajon Bank are a promising workaround to the issue of lack of governance – although this has limited success so far. The MPAs of Danajon Bank have been shown to support significantly different taxonomic and functional diversity to non-protected zones^64^, but the benefits of protected areas and marine no-take zones to community livelihoods are yet to be clearly demonstrated in order to encourage strong implementation locally.

## Discussion

### The land and sea routes ahead: ways forward and recommendations

All islands are vulnerable to climate change and its impacts, and to human activities that have led to the alarming loss of biodiversity. SIDS have emerged as representatives of the complex challenges of small island communities globally. They are important advocates and allies in finding solutions, and much has been accomplished by their collective voices. At the same time, it is important to recognize that small islands worldwide, not just SIDS, confront a wide range of challenges, particularly in terms of governance and the complex interplay between social and ecological factors.

Rather than seeing the focus on SIDS as disadvantageous, we acknowledge the shared vulnerability of all islands, and the similar but unique challenges they face in terms of conservation, climate change, and governance. We hope that the success of SIDS in highlighting their case can be built upon to open the discussion on the challenges of all islands around the world. This is important especially because the heterogeneity and diversity of small islands and their experiences remains poorly addressed by research and global policy. Urgent policy action, improved access to climate and biodiversity finance, and technical knowledge are essential for all small islands to mobilize the opportunities offered by the GBF and LDF.

In this context, we offer the following recommendations:

- Research is needed to broaden and also deepen the understanding of the unique challenges of islands beyond SIDS^4^, particularly around issues related to governance and policy. Research outcomes should directly inform policy. In particular, the heterogeneity of island challenges and opportunities should be explicitly recognized in major policy frameworks such as the UNFCCC and CBD and their relevant policy outcomes.
- A primary cause for gaps in knowledge in climate change impacts and adaptation is the unavailability of downscaled climate information that can be used for projecting future changes^2^, precisely because of the challenges posed by small islands’ size in climate models, a challenge also shared by geographically long and thin countries such as Chile. This is an area needs that active interest and support, in partnership with Indigenous and traditional knowledge-holders to overcome existing gaps in indigenous and local knowledge^45^, to develop local institutional capacities and address gaps in climate change adaptation.
- As the IAS target of the GBF is the only biodiversity target that specifically mentions islands, supporting initiatives towards island biodiversity monitoring should be considered an important component of IAS management and ecological restoration. This will help to establish baseline data that will be helpful in conservation, in addition to IAS management. Making these databases accessible for public use, research, and information are also important to address the general paucity of literature on island biodiversity, as this continues to be an impediment to global action.
- As small islands are marginalized systematically in development planning, funds or programs dedicated to island localities and organizations within archipelagic countries could assist in targeting resources for adaptation or natural resource management.
- Coalition-building and knowledge-sharing among SIDS and other island nations provides an opportunity to strengthen partnerships and learn from each other as a global, connected community.

Finally, all islands, including SIDS, represent both vulnerability and resilience. Embracing this duality and looking to small islands for leadership, including and especially from local, Indigenous and traditional knowledge, can help develop solutions that protect the diversity of nature’s values and human well-being for future generations on islands everywhere.
